# Supplementary material for: Barriers and facilitators to implementing social prescribing in child and adolescent mental health services: a qualitative analysis using the consolidated framework for implementation research
Source: Front Health Serv. 2026 Jul 8;6:1840754. doi: 10.3389/frhs.2026.1840754 (PMC13388805; doi:10.3389/frhs.2026.1840754)
Supplement: Supplementary file 1 [file Table1.docx]

| Table S1: Domain and construct definitions as per the CFIR 2.0 Outcomes Codebook |
| --- |
| Definitions |
| **1 Innovation (social prescribing): The “thing” being implemented, e.g., a new clinical treatment, educational program, or city service.** |
| **Evidence for innovation**: The degree to which the innovation has robust evidence supporting its effectiveness. |
| **Relative advantage**: The degree to which the innovation is better than other available innovations or current practice. |
| **Innovation design:** The degree to which the innovation is well designed and packaged, including how it is assembled, bundled, and presented. |
| **2 Outer setting: The setting in which the Inner Setting exists, e.g., hospital system, school district, state.** |
| **Local conditions**: The degree to which economic, environmental, political, and/or technological conditions enable the Outer Setting to support implementation and/or delivery of the innovation. |
| **Partnerships and connections**: The degree to which the Inner Setting is networked with external entities, including referral networks, academic affiliations, and professional organization networks. |
| **3 Inner setting (CAMHS): The setting in which the innovation is implemented, e.g., hospital, school, city.** |
| **Compatibility**: The degree to which the innovation fits with workflows, systems, and processes. |
| **Available resources**: The degree to which resources are available to implement and deliver the innovation. Note: Use this construct to capture themes related to Available Resources that are not included in the subconstructs below. |
| **4 Individuals: The roles and characteristics of individuals.** |
| **NHS-VCSE staff:**   - **Mid-level leaders:** Individuals with a moderate level of authority, including leaders supervised by a high-level leader and who supervise others. - **Implementation facilitators:** Individuals with subject matter expertise who assist, coach, or support implementation. |
| **Capability**: The degree to which these individuals have interpersonal competence, knowledge, and skills to fulfil Role. |
| **Link workers**   - **Innovation deliverers:** Individuals who are directly or indirectly delivering the innovation. |
| **Capability**: The degree to which innovation deliverers have interpersonal competence, knowledge, and skills to fulfil Role. |
| **5 Implementation process: The activities and strategies used to implement the innovation.** |
| **Assessing needs of deliverers:** The degree to which individuals collect information about the priorities, preferences, and needs of deliverers to guide implementation and delivery of the innovation. |
| **Assessing needs of recipients:** The degree to which individuals collect information about the priorities, preferences, and needs of recipients to guide implementation and delivery of the innovation. |
| **Engaging recipients / tailoring strategies** |
| The degree to which individuals attract and encourage recipients to serve on the implementation team and/or participate in the innovation  The degree to which individuals choose and operationalize implementation strategies to address barriers, leverage facilitators, and fit context. |
